# Dissemination project: Linking Clinical Practice and Research towards daily function and Mental Health prevention

**DOI:** 10.1192/j.eurpsy.2022.1763

**Published:** 2022-09-01

**Authors:** S. Regev, S. Rosenblum

**Affiliations:** 1 University of Haifa, Occupational Therapy, Haifa, Israel; 2 University of Haifa, Department Of Occupational Therapy Faculty Of Social Welfare & Health Sciences, Haifa, Israel

**Keywords:** Media, strategy, adhd, Occupational therapy

## Abstract

**Introduction:**

Occupational therapy (OT) offers evidence-based methods to address daily functioning improvement and better health promotion. However, many adults who are dealing with deficient daily functioning due to unrecognized Attention Deficit Hyperactivity disorder (ADHD) do not benefit from these developed methods. Thus, they are at risk of experiencing adjustment barriers and poor mental health.

**Objectives:**

The aim of this project is to develop a dissemination strategy in order to reach-out for this unrecognized ADHD population and grasp their attention in an early stage of life. The study following this project is measuring the possibility of this action to reach out to the individuals and make a small change in their daily functional capabilities.

**Methods:**

The Laboratory for the Study of Complex Human Activity and Participation (CHAP) is a lab sited in the occupational therapy department at the University of Haifa. Its research address ADHD as part of further neuro-developmental challenges in the life span. The lab started a dissemination program including 4 OT researchers, and a group of former researchers for materials feedback.

**Results:**

In the presentation we will share the dissemination strategy and it implementation during 6 months. Moreover, we will present a theoretically map relevant virtual pathways that adults with unrecognized ADHD may cross since childhood.

**Conclusions:**

This preventative program towards health promotion aims to help people achieve meaningful milestones in life and live more fully. Moreover, this strategy may serve as a prototype for similar approaches in other outreach processes.

**Disclosure:**

No significant relationships.

